# Violet Light Is Abundant Outdoors but Deficient Indoors in Modern Lifestyle in Tokyo

**DOI:** 10.3390/ijerph22030444

**Published:** 2025-03-17

**Authors:** Shinichiro Kondo, Xiaoyan Jiang, Hidemasa Torii, Kiwako Mori, Kazuno Negishi, Toshihide Kurihara, Kazuo Tsubota

**Affiliations:** 1 Tsubota Laboratory, Inc., Tokyo 160-8582, Japan; kondo@tsubota-lab.com; 2 Department of Ophthalmology, Keio University School of Medicine, Tokyo 160-8582, Japan; jxy@keio.jp (X.J.); htorii@keio.jp (H.T.); morikiwako@gmail.com (K.M.); kazunonegishi@keio.jp (K.N.); 3 Laboratory of Photobiology, Keio University School of Medicine, Tokyo 160-8582, Japan

**Keywords:** myopia, short-wavelength visible light, light environment, indoor lighting

## Abstract

This study examines the role of violet light (VL) in preventing myopia progression, addressing a critical need in urban environments where VL exposure is limited. Recent research suggests that VL, within the 360–400 nm wavelength range, may reduce myopia risk. To investigate, we conducted spectroscopic measurements in various settings across Tokyo, quantifying VL irradiance in natural sunlight. The results showed high VL levels outdoors, averaging 583 μW/cm^2^ on sunny days and 271 μW/cm^2^ on cloudy days, leading to a weighted annual average of approximately 310 μW/cm^2^. In contrast, indoor environments lacked VL due to UV-blocking materials in windows, glasses, and lighting. This deficiency may contribute to the rising incidence of myopia, particularly in urban areas with reduced outdoor exposure. Our findings highlight the need for innovative solutions to mitigate VL deficiency indoors, such as optimizing architectural designs and artificial lighting to better incorporate VL. This study provides foundational insights for future interventions aimed at reducing myopia risk through improved indoor light environments.

## 1. Introduction

Myopia has recently been focused on as a global health issue [1]. It used to be an issue for only a limited number of people half a century ago. However, in East Asian countries, the myopia population has grown fourfold in the last 60 years [2]. In those countries, the majority of children are myopic, as if it were a pandemic. Indeed, it is predicted that half of the world’s population will be myopic in 2050 [3].

Myopia occurs as the eyeball grows to the extent that the incident light is focused in front of the retina, resulting in blurred vision. With the excessive elongation of the eyeball’s axial length, a high degree of myopia is a risk factor for potential visual impairment, including blindness [4,5,6]. Although the cause of myopia has not been clearly identified, several strategies can be used against myopia [7]. Orthokeratology was invented and has been used in some countries. Orthokeratology hard contact lenses correct the shape of the corneal surface while sleeping, which in turn releases users from corrective devices during the day. Low-concentration atropine drops have been widely used for suppressive effects [8]. Clinical trials of the MiyoSmart contact lens by HOYA were completed, and the product entered the market in Hong Kong [9]. Clinical trials of the MiSight contact lens by Cooper Vision were completed, and the product is available in the U.S. and several other countries [10].

Various studies have largely agreed on the importance of the light environment [11]. The light intensity varies extensively in daily life from 0.1-lux-level moonlight [12] to up-to 100,000-lux-level sunlight. The human eye has a wide range of sensitivity, and one does not feel any inconvenience, whether outdoors or indoors. Rose et al. [11] proved that although near-work activities are normally considered negative to the eye, they have a smaller effect on the odds ratio of myopia as long as high levels of outdoor activities are maintained. From the extensive Recess Outside Classroom Trial 711 (ROCT711) program of Taiwan, a moderate amount of light intensity such as 1000 lux was found to be sufficient to show a protective effect against myopia with a longer duration than the brighter 10,000-lux case [13].

The Sun emits a broad spectrum of electromagnetic radiation, ranging from ultraviolet (UV) to infrared, with visible light spanning approximately 360 to 830 nm. The intensity distribution within this spectrum varies, with the highest energy concentrated in the visible and near-infrared ranges. In the Result section of this article, we illustrate the solar radiation spectra, highlighting the proportion of different wavelengths, including violet light (VL).

Violet light (VL) is part of the shortest-wavelength region of the visible spectrum, with wavelengths ranging from approximately 360 to 400 nm. While VL is present in natural sunlight, it is significantly reduced indoors due to modern architectural materials and lighting designs. According to the International Lighting Vocabulary by the Commission Internationale de l’Eclairage (CIE), the lower limit of visible light is defined as 360 nm [14]. Although VL falls within the visible spectrum, its perception is limited due to the human spectral luminous efficiency curve, which peaks in the green range and declines toward both spectral ends. Furthermore, the human cornea and lens exhibit low transmittance for wavelengths below 400 nm, a characteristic that becomes more pronounced with age [15,16]. As a result, only a small proportion of VL reaches the retina.

Beyond its role in vision, recent studies suggest that VL exposure influences circadian rhythm regulation, mood stabilization, and potential skin-related photobiological effects [17,18,19,20,21,22]. However, modern indoor environments significantly limit VL exposure due to the widespread use of UV-blocking materials, which may have broader physiological implications.

In addition to these general health effects, VL has been specifically implicated in the regulation of eye growth and myopia progression. In 2017, we reported that VL displayed a suppressive effect against myopia progression in chicks and humans [23]. Other studies have observed the same protective effect of VL in mice [24,25]. However, most prior studies have been conducted in controlled laboratory settings using animal models. While these studies provided valuable mechanistic insights, the extent to which VL exposure in real-world environments influences myopia progression remains unclear.

VL can be found in nature and in artificial lighting sources. Some materials transmit VL, whereas others do not. With a few examples, we have previously shown that VL exists abundantly outside in sunlight, whereas it is eliminated indoors in our modern lifestyle due to various so-called UV-protecting objects [23,26]. This study aims to systematically quantify VL availability in various indoor and outdoor settings, providing a comprehensive assessment of VL exposure under different environmental conditions and its implications for myopia prevention.

## 2. Materials and Methods

### 2.1. Measurement Devices Used

The spectral irradiance was measured using a fiber-optic spectrometer (BLUE-Wave, UVNb-50, StellarNet Inc., Tampa, FL, USA). The instrument had a 50 μm slit width, and the predicted resolution was 1.6 nm. The probe tip was installed with a light diffuser with a 180° field of view. The spectrometer and probe were calibrated for wavelength and intensity using the NIST-compliant method of the manufacturer. The spectrometer covers the wavelength range from UV to near-infrared. The measurement data were saved as a text file on a personal computer connected to the spectrometer. VL irradiance can be calculated by integrating the measured spectral irradiance in the VL wavelength range of 360–400 nm using the rectangular approximation.

The illuminance was measured using an illuminometer LX-1108 (KENIS LIMITED, Osaka, Japan). A dome-like light diffuser was used as the measurement probe. Data were recorded manually.

### 2.2. Measurements

For measurements, the probe was directed toward a light source or toward a certain direction, mimicking the human eye. For outdoor measurements, sunlight spectral irradiance data were collected at Keio University Shinanomachi Campus, Tokyo. Three different summer dates were chosen for the measurements without any particular intention, but the measurement time was all during the day when the Sun was high on the sky. The ground is a road paved with asphalt. The site was surrounded by buildings from which the horizon was not visible, which is a typical situation in Tokyo and other cities in developed countries. The weather was mostly cloudy on 5 July 2017, and the measurements were conducted at 14:30, 15:30, and 16:30 local time. It was again cloudy on the second day of 6 July 2017. The measurements were conducted three times in the morning at 10:00, 11:00, and 12:00. On the third day, 9 August 2017, it was mostly sunny with only a few clouds that did not block the Sun. On this day, four time slots, 11:00, 12:00, 13:00, and 14:00, were chosen for the measurements. For all sunlight measurements, the probe was pointed horizontally toward the four cardinal points, north, south, east, and west. Additionally, the Sun was measured with the probe pointed directly. On cloudy days, the Sun was behind the cloud, but the probe was directed toward the Sun.

While lighting fixtures were measured in our previous study [23], we measured two kinds of displays that are other representative emitting devices in this study. The iPhone (model: iPhone 6 plus) smartphone had a liquid-crystal display (LCD). The screen was set in white with 100% brightness and was measured by the probe 30 cm away from the display surface without any filter. The other display was LG’s organic light-emitting diode (OLED) television (TV) (model: OLED55C8PJA). The white screen was again measured at a distance of 30 cm.

In addition to emitting objects, we measured several transmitting objects with the Sun as the light source. As the two most representative ones, window glass and eyeglass lenses of different characteristics, but all clear in color, were chosen for spectral measurements. This is not a transmittance measurement of an object but a spectrum through a lens with the Sun as the lighting source. The indoor environment of a German automobile (BMW 435i) was measured through the front and side windows on a partly cloudy day on 11 June 2015, while it was being driven on a street in Tokyo, Japan. Indoor sunlight spectra were also measured on the same day in a modern condominium room in Tokyo through a window and lace curtain. School classrooms with windows of different transparent characteristics were measured. One school was a junior high school in Fujisawa, Kanagawa, Japan (measurement date: 13 April 2015), and the other was a kindergarten in Tokyo (measurement date: 16 December 2015). To study the position dependence of the incoming sunlight intensity from the window, both VL irradiance and illuminance were measured at equally spaced positions with the measuring probe pointing in the direction of the arrow. Note that fluorescent lights on the ceiling were kept on during the measurements in the classrooms.

Spectral irradiance was measured using three different eyeglass lenses on a cloudy day on 6 September 2017, in Tokyo. The three types of eyeglass lenses are a glass trial lens (Takagi Seiko Co., Ltd., Nagano, Japan), a plastic lens (JINS HOLDINGS Inc., Tokyo, Japan), and a VL-transmitting plastic lens (“JINS VIOLET+”, JINS HOLDINGS Inc., Tokyo, Japan).

## 3. Results

### 3.1. Outdoor Environment

**Figure 1:**
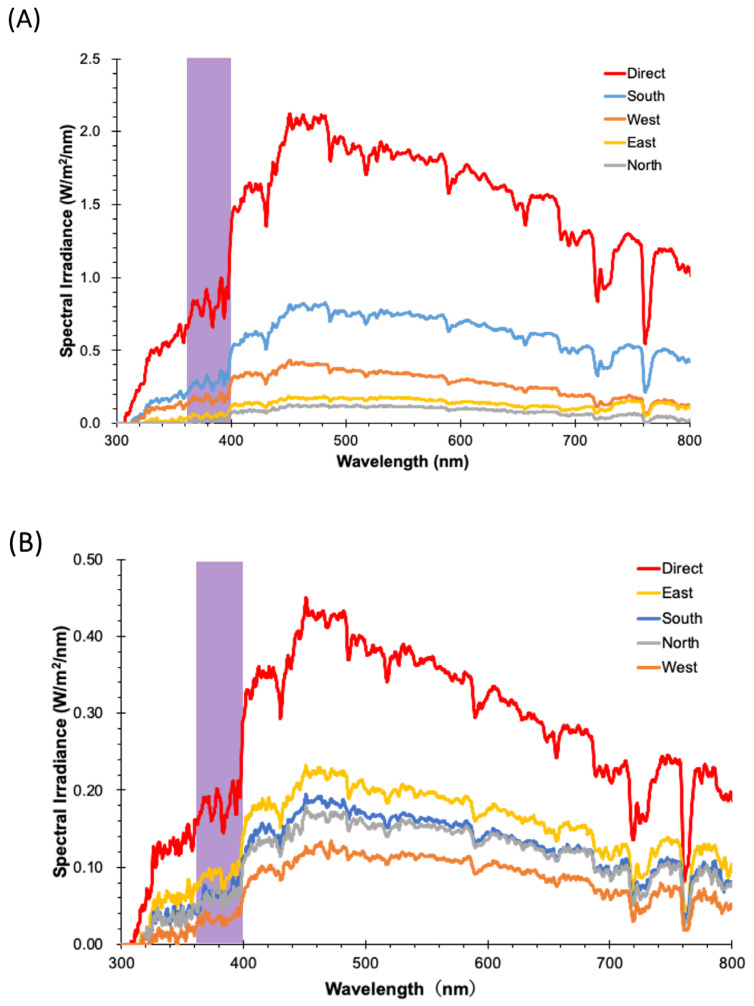
Sunlight spectra in the four directions as well as that toward the Sun (red) on (**A**) a sunny summer day at 13:00 and (**B**) a cloudy day at 12:00, both in Tokyo. Violet stripes illustrate the violet light (VL) region.

One of the three measurement days, 9 August 2017, was a clear, sunny day in Tokyo. The sunshine was intense and showed directional dependency, with the south as the strongest among the four directions from 11:00 to 13:00 when the Sun was high and not hidden by clouds. All measurement results are shown in Table 1 and in Figure S1. A representative set of sunny-day sunlight spectra measured at 13:00 is shown in Figure 1A. The VL irradiance in the southern direction was 1139 μW/cm^2^. However, the corresponding irradiance in the north direction was only 72 μW/cm^2^, which is more than 10 times smaller than that of the south. When the probe was directed toward the Sun directly, the VL irradiance was as high as 3000 μW/cm^2^ when the Sun was high, except at 14:00. From the measurement results of the four directions, the average VL irradiance during the measurement period was 583 μW/cm^2^.

The other two days were cloudy and the Sun was behind the clouds. However, they flowed constantly, varying the amount of incident sunlight through the clouds. A representative set of cloudy-day sunlight spectra measured at 12:00 on 6 July 2017 is shown in Figure 1B. The directional dependency was weaker on these two cloudy days, as shown in Table 2 below and Figures S2 and S3. Without direct sunlight, the measured spectra were obtained from diffused sunlight. The horizontal VL irradiance in the four directions was not systematic, but fluctuated as a result of constantly moving clouds. The incident sunlight was too dynamic to capture a representative spectrum at a given time and day. Any direction could have its maximum VL irradiance from 300 to 600 μW/cm^2^, whereas VL irradiance could be less than 100 μW/cm^2^ when thick clouds covered the Sun. Despite the cloudy sky and lack of direct sunshine, VL irradiance could exceed 1000 μW/cm^2^ when the probe was aimed at the Sun. However, when dense clouds momentarily covered the Sun, the measured VL irradiance became very low, such as 175 μW/cm^2^ at approximately 16:30 on 5 July 2017 (See Figure S2). From the measurement results of the four directions on these two days, the average VL irradiance during the measurement period was 271 μW/cm^2^.

Based on our limited number of outdoor measurements, we attempted to obtain an average VL irradiance of approximately 310 μW/cm^2^ in the following way. According to Japanese government statistics [27], there were 46 cloudless fine days in Tokyo in 2014. A “cloudless fine day” is defined in the statistics as the number of days when the amount of cloud is estimated below 1.5 in the 11-step evaluation method (0 minimum to 10 maximum amount of clouds) of the Japan Meteorological Agency. As described earlier in this section, we obtained 583 μW/cm^2^ as the average of VL irradiance for the four directions on a sunny day. We used this number for these 46 perfectly clear days in our calculations. For the remaining 319 days, we assumed that clouds existed in the sky. For these occasions, we used an average value of 271 μW/cm^2^ for cloudy days. From these assumptions, we obtained a weighted average of 310 μW/cm^2^, as shown in the following equation:

$$583\frac{\mu W}{{cm}^{2}}\times \frac{46\;days}{365\;days}+271\frac{\mu W}{{cm}^{2}}\times \frac{319\;days}{365\;days}=310\frac{\mu W}{{cm}^{2}}$$

This is an approximate calculation based on simple statistics and a limited amount of observed data.

### 3.2. Indoor Environment

#### 3.2.1. Display

**Figure 2:**
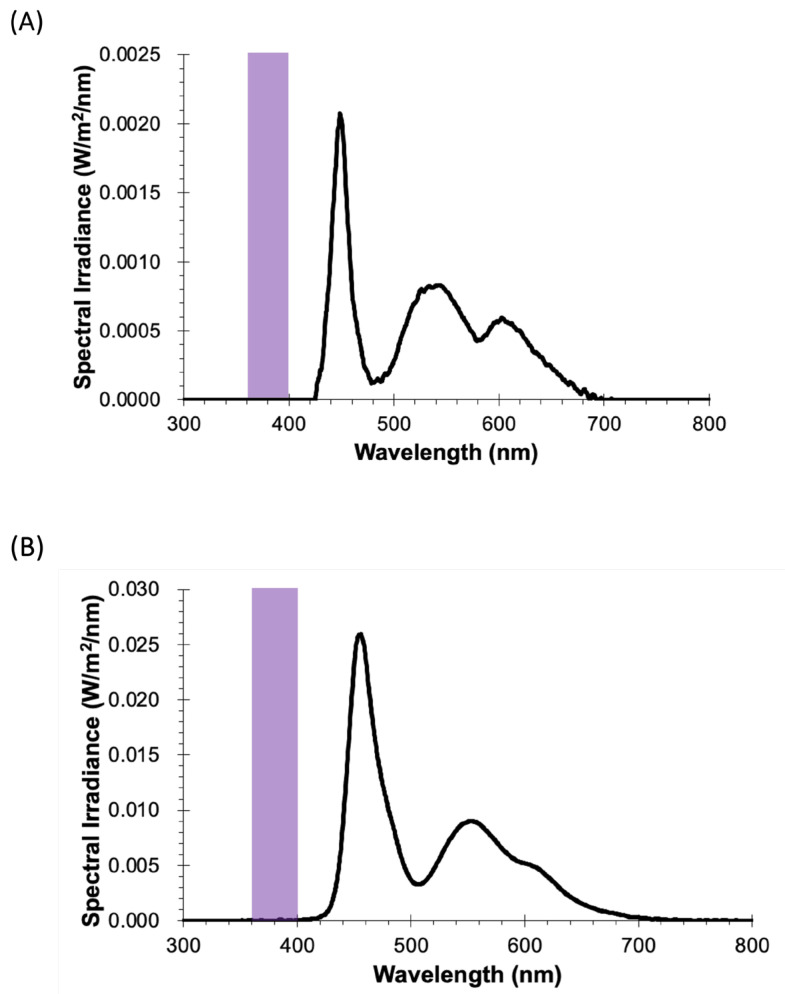
Emission spectra (black) of 2 different types of displays: (**A**) smartphone and (**B**) organic light-emitting-diode (OLED) display, both measured at 30 cm from the screen. Violet stripes illustrate the violet light (VL) region.

The emission spectra for the two types of modern displays, LCD and OLED, measured at a 30 cm distance, are shown in Figure 2A,B. The spectra for the white screen are similar in peak composition, consisting of blue, green, and orange-to-red peaks. No VL component was observed in either of the spectra. The smartphone LCD had one-tenth of the light intensity of the OLED TV.

#### 3.2.2. Automobile Windows

**Figure 3:**
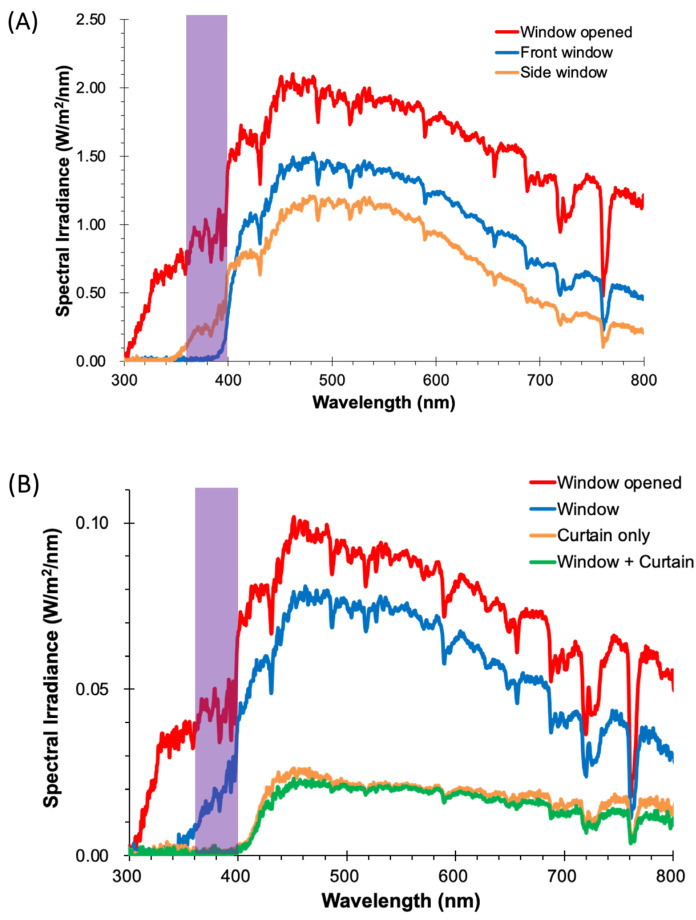
Sunlight spectra through automobile and room windows: (**A**) spectra of automobile front window (blue), side window (orange), and window opened (red); (**B**) spectra in a private room with window opened (red) and through window (blue), curtain only (orange) and through window and curtain (green).

Figure 3A shows the transmittance characteristics of automobile windows with respect to the Sun. The jagged but continuous sunlight spectrum, represented in red, continued to show finite spectral irradiance values down to approximately 300 nm. The front window eliminated sunlight with wavelengths of up to approximately 400 nm. Meanwhile, the side window had a different filtering property from that of the front window, with a cut-off wavelength of 350 nm.

#### 3.2.3. Window in a Condominium Room

As Figure 3B shows in blue, the window in the condominium room blocked sunlight below 350 nm, similarly to the side automobile window shown in Figure 3A. Moreover, there was a curtain just inside the window, which not only raised the cut-off wavelength to 400 nm, but also reduced the transmitted intensity of the incoming sunlight inside the room.

#### 3.2.4. Classroom Windows

**Figure 4:**
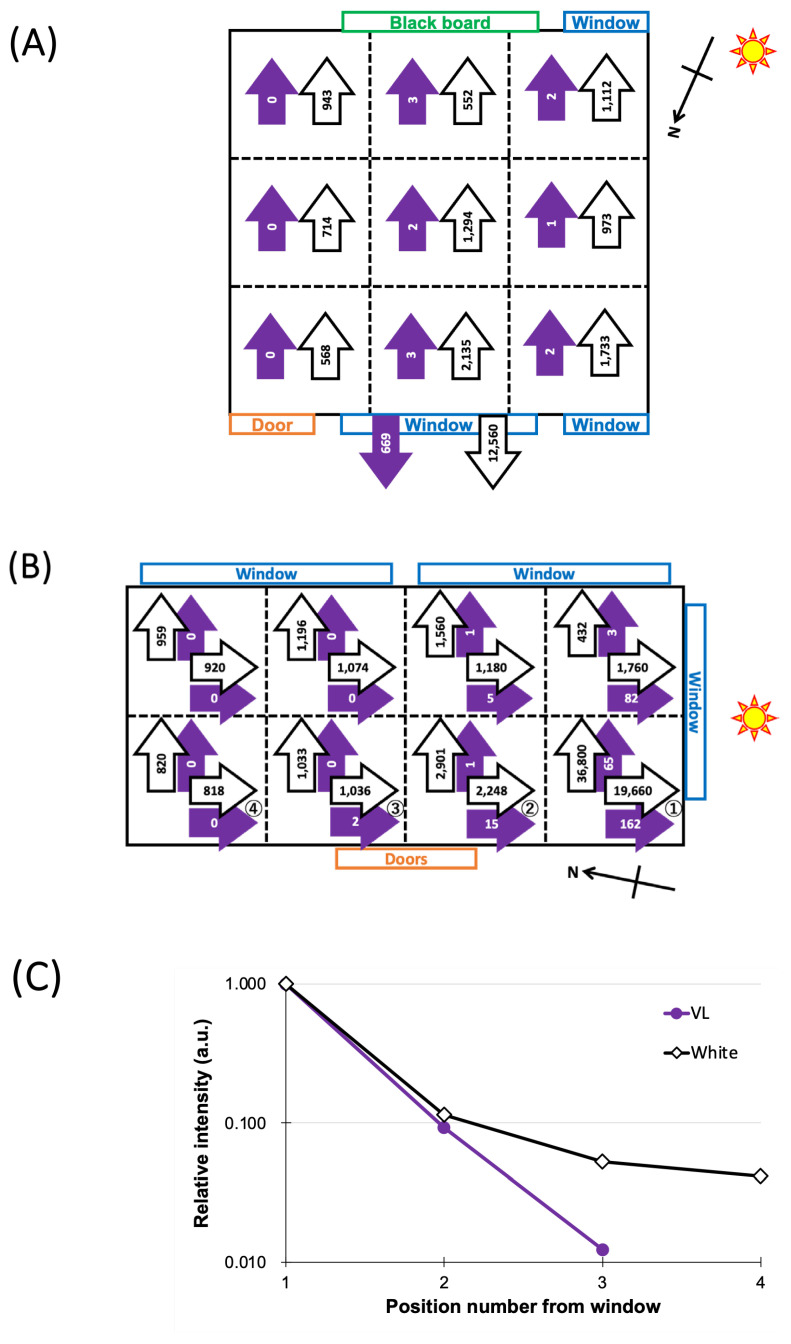
Sunlight violet light (VL) irradiance (violet arrows in µW/cm^2^) and illuminance (white arrows in lux) measured in different positions and directions inside classrooms: (**A**) a junior high school classroom with VL-blocking windows; (**B**) a kindergarten classroom with VL-transmitting windows, where positions ① to ④ indicate different measurement locations inside the classroom, with position ① closest to the window and position ④ farthest from it. Positions ① to ④ correspond to measurement locations along the x-axis in (**C**).; (**C**) Sunlight intensity (VL irradiance and illuminance) plotted against measurement positions 1 to 4, normalized to position ①.

In the junior high school classroom, just-outside values over the rear-side window were also measured. As Figure 4A shows, both VL irradiance and illuminance were found to be much greater than those indoors. The indoor VL irradiance values were negligibly small, irrespective of the position, because of the VL-blocking window glass. On the other hand, the measured illuminance across the classroom showed moderate values of 500–1000 lux throughout the classroom.

The window glass of the kindergarten classroom allowed VL to be transmitted inside the room. Figure 4B illustrates how the incoming VL rapidly diminished as the measurement positions were farther away from the window situated in the direction of the Sun. As in Figure 4C, the measured illuminance decreased in a manner similar to the VL irradiance.

#### 3.2.5. Eyeglass Lenses

**Figure 5:**
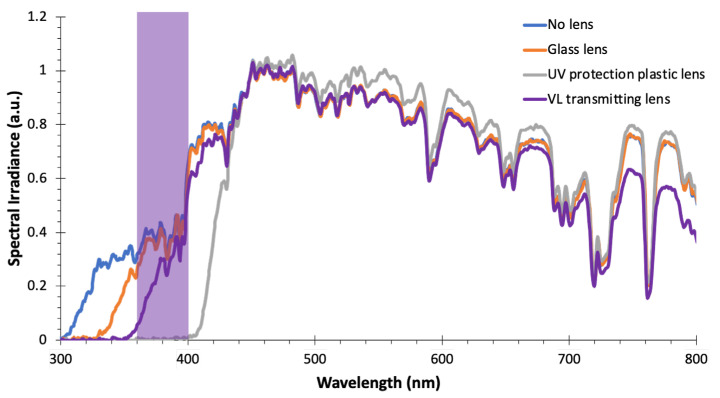
Sunlight spectra of eyeglass lenses: without lens (blue) and through three different types of lenses: glass lens (orange), UV-protection plastic lens (gray), and VL-transmitting lens (violet). VL irradiance are 71.27, 63.36, 0.65, and 46.20 µW/cm^2^, respectively.

Figure 5 compares the different types of eyeglass lenses, including the “no lens” case that is simply the sunlight spectrum itself. The short-wavelength part of the sunlight spectrum was eliminated to different extents depending on the lens type. A glass trial lens was used instead of a real eyeglass lens, as it is practically impossible to find glass-made eyeglass lenses. It blocks sunlight below 330 nm. Plastic eyeglass lenses have become the standard in modern society. The measured plastic lens blocked sunlight to below 400 nm. The third type of measured lens was the JINS VIOLET+. It has been commercially available in Japan since summer 2017. JINS VIOLET+ lenses allow VL to transmit while still blocking light below 350 nm, as shown by the purple line in Figure 5.

## 4. Discussion

The Sun is the ultimate lighting source. As detected in our outdoor measurements, sunlight has a broad spectrum, ranging from UV-B to infrared, including VL, and is very intense. The Sun delivers sufficient energy to the ground. By integrating the spectrum for all wavelengths, one can find the order of 100 k μW/cm^2^ sunlight power delivered to the ground. VL, defined for wavelengths from 360 to 400 nm, occupies only approximately 3% of the power of the entire spectrum. Facing the Sun directly at midday when there were no clouds, 3000 μW/cm^2^ or more VL was obtained, as shown in Figure S1. However, for safety reasons, we normally do not look up at the Sun, keeping the line of sight approximately horizontal, which limits the incident VL that enters the eye.

Our previous study [23] showed that VL is abundant outdoors but significantly reduced indoors due to UV-blocking materials, with negligible exposure in windowless rooms and very low levels even with windows. However, it did not consider the impact of directional factors on VL exposure. In contrast, our current study systematically quantifies VL in various real-life settings, including classrooms, and examines the influence of directional factors (north, south, east, and west). These findings highlight how VL availability depends not only on indoor conditions but also on external environmental factors, further emphasizing the severe deficiency of VL in modern indoor settings.

The effects of VL exposure on myopia suppression have been studied in both animal models and human clinical trials. Jeong et al. [28] demonstrated in a murine myopia model that VL transmission ratios influenced myopia progression, with significant suppression observed at VL transmittance levels of 70% and above, whereas 40% transmittance showed only minor effects. This suggests a potential threshold for VL exposure necessary to elicit a biological response in myopia control. In human studies, a randomized controlled trial by Mori et al. [29] demonstrated that VL-transmitting eyeglasses significantly reduced axial elongation over a two-year period, with no reported adverse effects. This suggests that moderate VL exposure is not only effective in suppressing myopia progression but also safe for long-term use. Given the substantial reduction in VL indoors due to modern architectural materials, future studies should aim to determine the minimum effective dose of VL required to achieve meaningful myopia suppression in humans while ensuring safety.

The average VL irradiance of 310 μW/cm^2^ obtained in the previous section was called a yearly average, to the extent that the weather statistics used was a yearly figure. However, as there is no seasonal information, it is not ideal to combine these statistics with the underlying measurement results of only summer. The average also presents a fundamental difficulty with the measurement scheme. The amount of measurement data should be sufficiently large, but the sky is too dynamic to capture the state of the moment. Consequently, irradiation can constantly change at any given moment. The average irradiance could also be found from continuous measurements over time by dividing the irradiated energy by the exposure time. If this type of continuous measurement is repeated over a long period of time for different seasons of the year, the quality of the average figure becomes better and closer to the true yearly average. The measurement location was just one place in Tokyo. Thus, the results might not be applicable in other parts of the world. Our current results are not conclusive at least quantitatively, requiring us to conduct further studies for statistically better results. Nevertheless, they should well reflect the circumstances of similar developed countries.

In our modern lifestyle, there are almost no artificial lighting sources that provide VL, with a few exceptions. Following the first-generation artificial lighting source of candles, the incandescent lamp from the last century is called the second-generation household lamp, and it has low VL [23]. Fluorescent lamps (third generation) and LED lights (fourth generation) have no VL included in their emitted white light. Therefore, if we were to take VL indoors, we must make the VL component of the sunlight come indoors, or an artificial VL lighting source such as a VL LED should be used.

Our modern lifestyle is surrounded by various so-called “UV-protecting” items that block not only ultraviolet light, but also VL. These are high-pass filters, leaving out the low-wavelength components. Visible light transmits these UV-protecting items, thereby producing white light. As far as types of window glass are concerned, relatively old and new UV-protecting window glass coexist in our current society. The old and simple type of window glass permits VL transmission, and it is still being used in buildings equipped with “standard” glass. Nevertheless, the trend toward UV protection has accelerated. UV protection is an additional added value to special interior spaces. For example, our previous study reported a sharp drop in sunlight spectral irradiance at 400 nm in motor vehicles and modern buildings [23]. In contrast, the sunlight component down to a wavelength of 350 nm, including VL, penetrated through the side window of the vehicle (orange line in Figure 3A) and also through the condominium room window (blue line in Figure 3B). Over such a window, the curtain provided extra UV protection in the room, increasing the cut-off wavelength to 400 nm, and therefore transmitting no VL indoors.

In addition, other UV-protecting items that can be found in society are lenses to the eye. Current eyeglass lenses are made of plastic, and with extra surface coating, the so-called UV400 requirement is met [30]. Figure 5 clearly indicates that the short-wavelength end of the sunlight spectrum is truncated by the different types of eyeglass lenses. A glass lens with a cut-off wavelength of 330 nm was used only as a trial lens and not for eyeglasses. A UV400 lens is a typical choice for current users. The VL transmitting lens was situated between these two lenses.

It has been recently shown that VL, the shortest-wavelength visible light, plays an important role in protecting the eye from myopic progression [23,26]. There is unfortunate bias and confusion surrounding the term ultraviolet (UV), as it is often used based on convention rather than strict optical visibility. The most natural definition of UV is that it begins where light becomes invisible as its wavelength decreases. Following this principle, the shortest visible light is violet (VL), and the adjacent, non-visible range is ultraviolet (UV). The Commission Internationale de l’Eclairage (CIE), which formally defines visible light, states that the shortest-wavelength visible radiation extends down to 360 nm [14].

However, another classification—widely used in safety standards—defines UV as encompassing wavelengths up to 400 nm, dividing it into three subcategories: UV-A (315–400 nm), UV-B (280–315 nm), and UV-C (below 280 nm) [31]. This classification is the basis for the widely known UV400 standard and many UV-blocking materials, which often filter out not only UV but also a portion of VL [30].

From a photobiological safety perspective, the International Commission on Non-Ionizing Radiation Protection (ICNIRP) provides guidelines [32] for exposure limits to ultraviolet radiation, including UV-A (315–400 nm). These guidelines are designed to ensure safety against acute phototoxic effects, though they do not directly address the chronic effects of long-term exposure. Importantly, while VL is defined by CIE [14] as the shortest-wavelength visible light (360–400 nm), ICNIRP classifies VL as falling within the UV-A range based on its wavelength range rather than its visibility. Although our estimated VL irradiance is significantly lower than ICNIRP’s recommended exposure limits for UV radiation, prolonged direct sun exposure, particularly in extreme conditions, may pose different risks and should be considered separately.

An additional interesting characteristic was found in our school-classroom measurements. The window glass was all UV-protected in junior high schools. Only negligibly small or zero VL values were found indoors. As depicted in Figure 4A, the illuminance measured in the front direction came from the fluorescent lamps on the ceiling and the Sun through two windows located at the front and back of the classroom. In contrast, the kindergarten was equipped with old-fashioned window glass, which allowed VL to enter the classroom. However, as illustrated in Figure 4B,C, the VL power measured at different positions inside the classroom decreased quickly as it was farther from the window, with the Sun present outside at the time of measurements. The measured illuminance behaved similarly in the same classroom, decreasing according to the distance from the window, in the direction of the Sun. The illuminance was again measured for the two white light sources, as in the junior high school case. As VL irradiance decreased, the measured illuminance also decreased. The room was kept sufficiently bright in terms of the illuminance. However, we did not recognize the rapidly diminishing VL or even the presence or absence of VL in the room because of the low VL sensitivity of the eye.

## 5. Conclusions

Despite the widely varying weather and consequent change in intensity, the brightness of sunlight is normally much stronger than that of artificial lighting sources, such as lamps and displays. VL is abundant outdoors under sunlight. An average VL irradiance of 310 μW/cm^2^ was determined using a weighted calculation under basic assumptions. Exposing the eye to this much VL power for two or three hours could prevent myopic eyes from worsening.

Once one goes indoors, VL can hardly be found, except near the window with VL-transmitting glass, because of the prevalent UV-protecting items. Moreover, among the ordinary household lighting sources currently available, there are no VL-emitting lighting sources. Therefore, one must rely on sunlight as the source of VL, and to have the naked eye receive or wear VL-transmitting lenses to receive VL.

Given these findings, future efforts should focus on designing architectural solutions that allow for greater VL transmission indoors, such as optimized window coatings and specialized daylighting strategies. Additionally, advancements in artificial lighting technology, including VL-emitting LEDs, could help compensate for the lack of natural VL exposure in indoor environments. These innovations could play a crucial role in mitigating VL deficiency and potentially contribute to myopia prevention.

If VL-transmitting and VL-emitting items are to be developed, their safety and efficacy should be thoroughly studied for potential use in myopia prevention.

## Figures and Tables

**Table 1:** Measurement results and calculated average values of violet light (VL) irradiance on a sunny day.

VL Irradiance (µW/cm^2^)						
Date	Time	East	South	West	North	Sun
**Sunny day on 9 August 2017**	11:00	806	874	264	297	3480
	12:00	19	1754	746	164	3552
	13:00	210	1139	672	72	3296
	14:00	177	790	1154	195	2306
**Average**		583				3159

**Table 2:** Measurement results and calculated average values of violet light (VL) irradiance on two cloudy days.

VL Irradiance (µW/cm^2^)						
Date	Time	East	South	West	North	Sun
**Cloudy day on 6 July 2017**	10:00	177	381	265	363	1160
	11:00	352	459	312	610	1674
	12:00	363	262	138	250	737
**Cloudy day on 5 July 2017**	14:30	283	313	489	410	1158
	15:30	147	94	161	351	660
	16:30	66	72	62	115	175
**Average**		271				927

## Data Availability

Additional data are available from the corresponding authors on request.

## Supplementary Materials

The following supporting information can be downloaded at: https://www.mdpi.com/article/10.3390/ijerph22030444/s1, Figure S1: Sunlight spectral irradiance in all five directions for all measurement times on a sunny day on 9 August 2017, Figure S2: Sunlight spectral irradiance in all five directions for all measurement times on a cloudy day on 5 July 2017, Figure S3: Sunlight spectral irradiance in all five directions for all measurement times on a mostly cloudy day on 6 July 2017.

## References

[1] World Health Organization (2017). The Impact of Myopia and High Myopia.

[2] Dolgin E. (2015). The myopia boom. Nature.

[3] Holden B.A., Fricke T.R., Wilson D.A., Jong M., Naidoo K.S., Sankaridurg P., Wong T.Y., Naduvilath T.J., Resnikoff S. (2016). Global Prevalence of Myopia and High Myopia and Temporal Trends from 2000 through 2050. Ophthalmology.

[4] Ikuno Y. (2017). Overview of the Complications of High Myopia. Retina.

[5] Ohno-Matsui K. (2016). Pathologic Myopia. Asia Pac. J. Ophthalmol..

[6] Ohno-Matsui K., Lai T.Y.Y., Lai C.-C., Cheung C.M.G. (2016). Updates of pathologic myopia. Prog. Retina Eye Res..

[7] Brennan N.A., Toubouti Y.M., Cheng X., Bullimore M.A. (2020). Efficacy in myopia control. Prog. Retina Eye Res..

[8] Chia A., Lu Q.S., Tan D. (2016). Five-Year Clinical Trial on Atropine for the Treatment of Myopia 2: Myopia Control with Atropine 0.01% Eyedrops. Ophthalmology.

[9] Lam C.S.Y., Tang W.C., Tse D.Y., Lee R.P.K., Chun R.K.M., Hasegawa K., Qi H., Hatanaka T., To C.H. (2020). Defocus Incorporated Multiple Segments (DIMS) spectacle lenses slow myopia progression: A 2-year randomised clinical trial. Br. J. Ophthalmol..

[10] Chamberlain P., Peixoto-de-Matos S.C., Logan N.S., Ngo C., Jones D., Young G. (2019). A 3-year Randomized Clinical Trial of MiSight Lenses for Myopia Control. Optom. Vis. Sci..

[11] Rose K.A., Morgan I.G., Ip J., Kifley A., Huynh S., Smith W., Mitchell P. (2008). Outdoor activity reduces the prevalence of myopia in children. Ophthalmology.

[12] Kyba C., Mohar A., Posch T. (2017). How bright is moonlight?. Astron. Geophys..

[13] Wu P.C., Chen C.T., Lin K.K., Sun C.C., Kuo C.N., Huang H.M., Poon Y.C., Yang M.L., Chen C.Y., Huang J.C. (2018). Myopia Prevention and Outdoor Light Intensity in a School-Based Cluster Randomized Trial. Ophthalmology.

[14] Commission Internationale de l’Eclairage (CIE) (2020). e-ILV (International Lighting Vocabulary): Visible Radiation. https://cie.co.at/eilvterm/17-21-003.

[15] Artigas J.M., Felipe A., Navea A., Fandiño A., Artigas C. (2012). Spectral Transmission of the Human Crystalline Lens in Adult and Elderly Persons: Color and Total Transmission of Visible Light. Investig. Ophthalmol. Vis. Sci..

[16] Sliney D.H. (2002). How light reaches the eye and its components. Int. J. Toxicol..

[17] Buhr E.D., Vemaraju S., Diaz N., Lang R.A., Van Gelder R.N. (2019). Neuropsin (OPN5) Mediates Local Light-Dependent Induction of Circadian Clock Genes and Circadian Photoentrainment in Exposed Murine Skin. Curr. Biol..

[18] Buhr E.D., Yue W.W., Ren X., Jiang Z., Liao H.W., Mei X., Vemaraju S., Nguyen M.T., Reed R.R., Lang R.A. (2015). Neuropsin (OPN5)-mediated photoentrainment of local circadian oscillators in mammalian retina and cornea. Proc. Natl. Acad. Sci. USA.

[19] Díaz N.M., Lang R.A., Van Gelder R.N., Buhr E.D. (2020). Wounding Induces Facultative Opn5-Dependent Circadian Photoreception in the Murine Cornea. Investig. Ophthalmol. Vis. Sci..

[20] Nguyen M.T., Vemaraju S., Nayak G., Odaka Y., Buhr E.D., Alonzo N., Tran U., Batie M., Upton B.A., Darvas M. (2019). An opsin 5-dopamine pathway mediates light-dependent vascular development in the eye. Nat. Cell Biol..

[21] Ota W., Nakane Y., Hattar S., Yoshimura T. (2018). Impaired Circadian Photoentrainment in Opn5-Null Mice. iScience.

[22] Zhang K.X., D’Souza S., Upton B.A., Kernodle S., Vemaraju S., Nayak G., Gaitonde K.D., Holt A.L., Linne C.D., Smith A.N. (2020). Violet-light suppression of thermogenesis by opsin 5 hypothalamic neurons. Nature.

[23] Torii H., Kurihara T., Seko Y., Negishi K., Ohnuma K., Inaba T., Kawashima M., Jiang X., Kondo S., Miyauchi M. (2017). Violet Light Exposure Can Be a Preventive Strategy Against Myopia Progression. EBioMedicine.

[24] Jiang X., Pardue M.T., Mori K., Ikeda S.I., Torii H., D’Souza S., Lang R.A., Kurihara T., Tsubota K. (2021). Violet light suppresses lens-induced myopia via neuropsin (OPN5) in mice. Proc. Natl. Acad. Sci. USA.

[25] Strickland R., Landis E.G., Pardue M.T. (2020). Short-Wavelength (Violet) Light Protects Mice From Myopia Through Cone Signaling. Investig. Ophthalmol. Vis. Sci..

[26] Torii H., Ohnuma K., Kurihara T., Tsubota K., Negishi K. (2017). Violet Light Transmission is Related to Myopia Progression in Adult High Myopia. Sci. Rep..

[27] Statistics Bureau of Japan (2014). System of Social and Demographic Statistics. https://www.e-stat.go.jp/en/stat-search/file-download?statInfId=000023621249&fileKind=0.

[28] Jeong H., Kurihara T., Jiang X., Kondo S., Ueno Y., Hayashi Y., Lee D., Ikeda S.I., Mori K., Torii H. (2023). Suppressive effects of violet light transmission on myopia progression in a mouse model of lens-induced myopia. Exp. Eye Res..

[29] Mori K., Torii H., Hara Y., Hara M., Yotsukura E., Hanyuda A., Negishi K., Kurihara T., Tsubota K. (2021). Effect of Violet Light-Transmitting Eyeglasses on Axial Elongation in Myopic Children: A Randomized Controlled Trial. J. Clin. Med..

[30] (2018). Ophthalmic Optics—Spectacle Lenses—Short Wavelength Visible Solar Radiation and the Eye.

[31] Commission Internationale de l’Eclairage (CIE) (2020). e-ILV (International Lighting Vocabulary): Ultraviolet Radiation. https://cie.co.at/eilvterm/17-21-008.

[32] International Commission on Non-Ionizing Radiation Protection (2004). Guidelines on limits of exposure to ultraviolet radiation of wavelengths between 180 nm and 400 nm (incoherent optical radiation). Health Phys..

