# Supplementary material for: Violet Light Is Abundant Outdoors but Deficient Indoors in Modern Lifestyle in Tokyo
Source: Int J Environ Res Public Health. 2025 Mar 17;22(3):444. doi: 10.3390/ijerph22030444 (PMC11942253; doi:10.3390/ijerph22030444)
Supplement: Supplementary file 1 [file ijerph-22-00444-s001.zip › ijerph-3457504-supplementary.pdf]

International Journal of
Environmental Research and Public Health

Supplementary figures

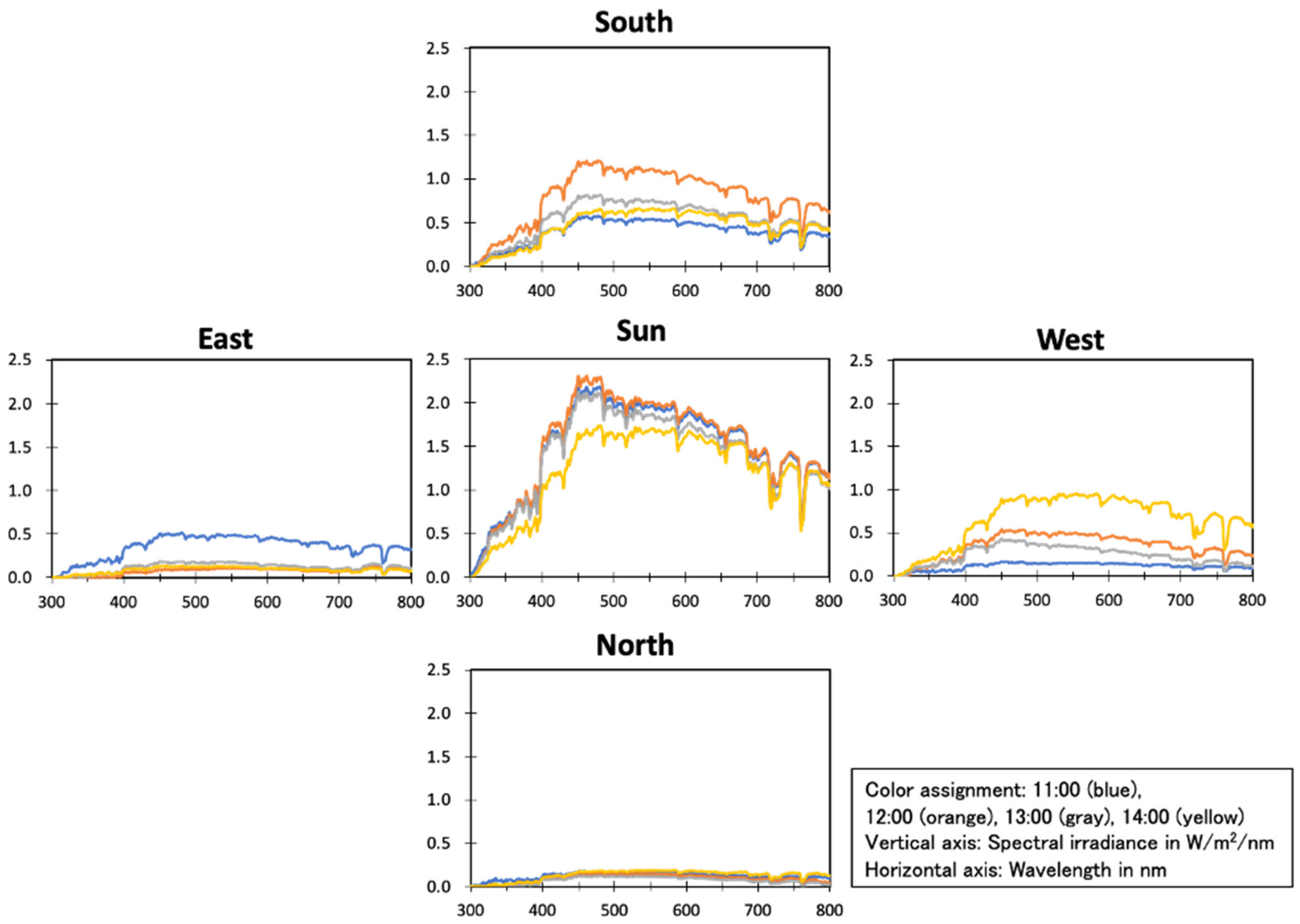

Figure S1. Sunlight spectral irradiance in all five directions for all measurement times on a sunny day on August 9, 2017.

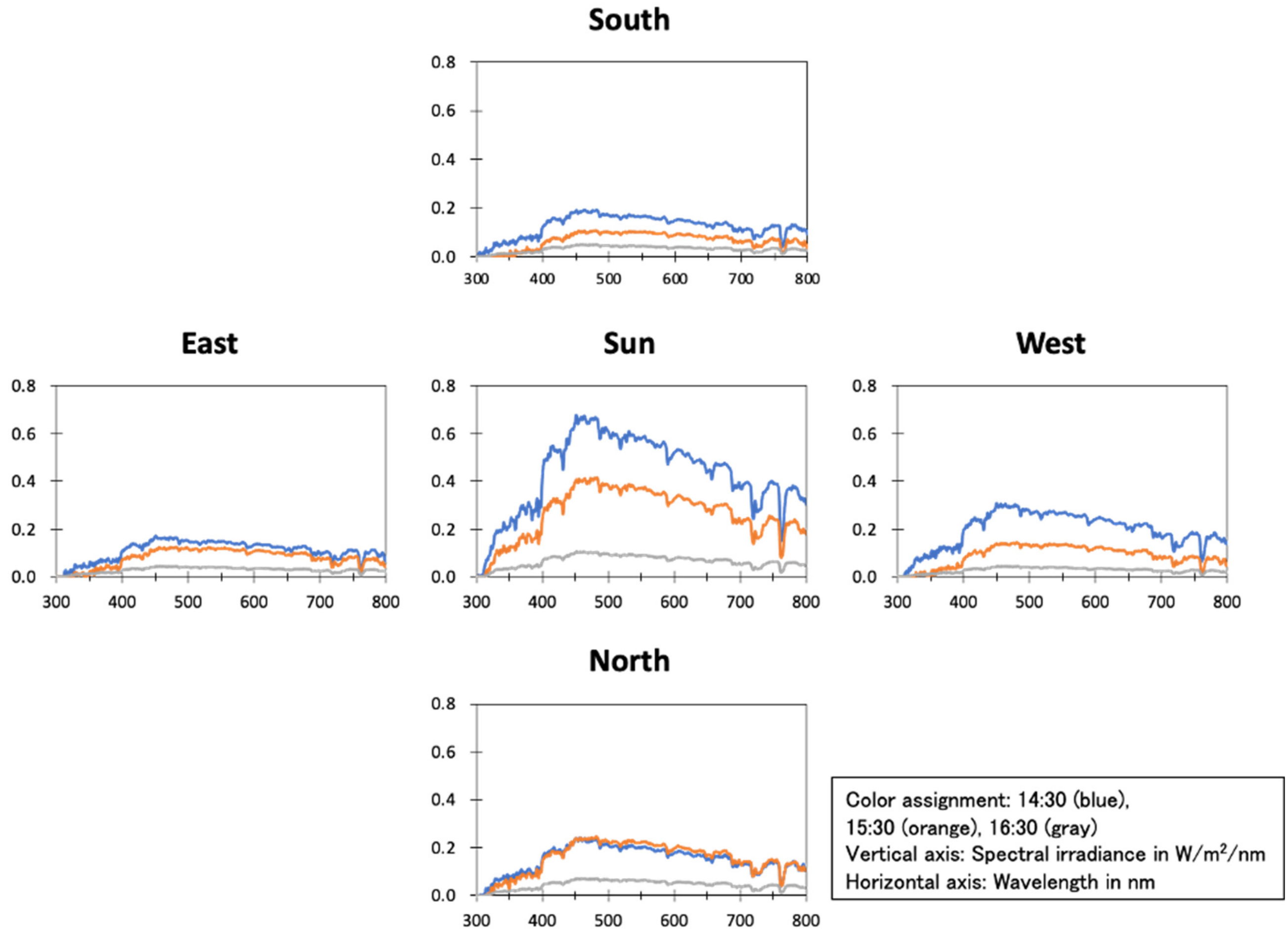

Figure S2. Sunlight spectral irradiance in all five directions for all measurement times on a cloudy day on July 5, 2017.

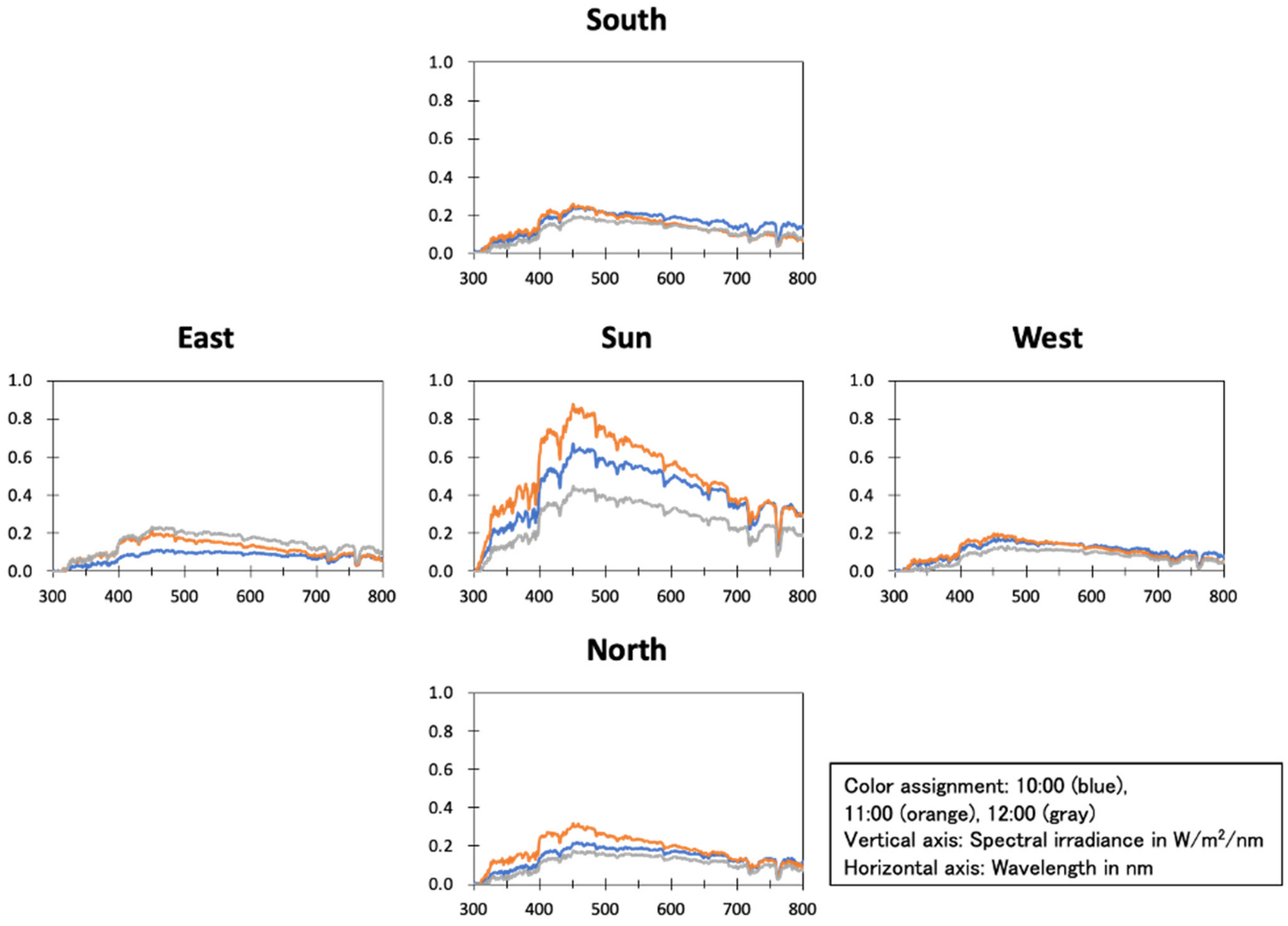

Figure S3. Sunlight spectral irradiance in all five directions for all measurement times on a mostly cloudy day on July 6, 2017.

Disclaimer/Publisher's Note: The statements, opinions and data contained in all publications are solely those of the individual author(s) and contributor(s) and not of MDPI and/or the editor(s). MDPI and/or the editor(s) disclaim responsibility for any injury to people or property resulting from any ideas, methods, instructions or products referred to in the content.
